# The rs3844492/*ARHGAP22* and rs741301/*ELMO1* polymorphisms are associated with changes in laboratory markers of renal damage among patients with type 2 diabetes mellitus

**DOI:** 10.20945/2359-4292-2024-0167

**Published:** 2025-04-18

**Authors:** Luciane Moretto, Eliandra Girardi, Anna Carolina Meireles Vieira, Letícia de Almeida Brondani, Natália Emerim Lemos, Luís Henrique Canani, Marilu Fiegenbaum, Cristine Dieter, Daisy Crispim

**Affiliations:** 1 Serviço de Endocrinologia, Hospital de Clínicas de Porto Alegre, Porto Alegre, RS, Brasil; 2 Programa de Pós-Graduação em Ciências Médicas: Endocrinologia, Faculdade de Medicina, Departamento de Medicina Interna., Universidade Federal do Rio Grande do Sul, Porto Alegre, RS, Brasil; 3 Universidade Federal de Ciências da Saúde de Porto Alegre, Porto Alegre, RS, Brasil; 4 Unidade de Pesquisa Laboratorial, Centro de Pesquisa Experimental Hospital de Clínicas de Porto Alegre, Porto Alegre, RS, Brasil; 5 Departamento de Bioquímica, Instituto de Química, Universidade de São Paulo, São Paulo, SP, Brasil

**Keywords:** Diabetes mellitus, Diabetic nephropathies, Polymorphism, genetic

## Abstract

**Objective:**

To investigate the association between the rs3844492/*ARHGAP22* and rs741301/*ELMO1* polymorphisms and diabetic kidney disease in patients with type 2 diabetes mellitus.

**Methods:**

The sample consisted of 740 patients with type 2 diabetes mellitus and diabetic kidney disease (cases) and 303 patients with type 2 diabetes mellitus, but no diabetic kidney disease (controls). The genotyping of the polymorphisms was conducted using real-time polymerase chain reaction with Taqman probes.

**Results:**

The frequency of the rs3844492/ARHGAP22 G/G genotype was 16.8% in the control group and 15.7% in cases (p = 0.069). After adjusting for covariables, the presence of the G allele was associated with risk for diabetic kidney disease (OR = 1.435, 95% CI 1.023 – 2.011; p = 0.036), as well as with a decreased estimated glomerular filtration rate (p = 0.012) and elevated creatinine levels (p = 0.009). No difference was observed in the rs741301/ELMO1 genotype frequencies between groups (p = 0.800). However, the presence of the C allele appears to be associated with higher creatinine levels in patients with type 2 diabetes mellitus (p = 0.064).

**Conclusion:**

The rs3844492/*ARHGAP22* and rs741301/*ELMO1* polymorphisms are associated with alterations in renal function markers among patients with type 2 diabetes mellitus.

## INTRODUCTION

Diabetic kidney disease (DKD) is an important microvascular complication, impacting 40% of patients with diabetes mellitus (DM) (^1^). Globally, it is recognized as the primary cause of chronic kidney disease (CKD) and end-stage chronic renal disease (ESRD), emerging as a major predictor of mortality in patients with DM (^2^). The manifestation of DKD typically depends on the duration of DM, patient age, systemic arterial hypertension, dyslipidemia, and glycemic control. Clinically, DKD is characterized by the presence of albuminuria and/or a progressive decline in glomerular filtration rate (GFR) (^3^).

It is widely acknowledged that, in addition to the conventional environmental risk factors previously outlined, a robust genetic component significantly influences the onset of DKD (^4,5,6,7^). Several genome-wide association studies (GWAS) have pinpointed various susceptibility *loci* contributing to the development or progression of microvascular complications of DM (^8,9,10,11^). Among these *loci,* the *ARHGAP22* and *ELMO1* genes are potential candidate genes for DKD due to their roles in pathways related to pathogenesis of DM complications (^12,13^).

The *Rho GTPase activating protein 22 (ARHGAP22)* gene, situated on chromosome 10q11.22 (^14^), encodes the Rho protein, a member of the GTPase-activating protein family (^15^). This protein family plays a pivotal role in various cellular processes, including cell motility, angiogenesis, and increased cell permeability (^14,16^). Li and cols. (^17^) demonstrated that polymorphisms in the *ARHGAP22* gene are associated with risk for type 2 DM (T2DM) and suggested this gene may also have a role as an insulin regulator. Moreover, the involvement of *ARHGAP22* in the mobilization or active circulation of endothelial progenitor cells (EPCs) has been proposed, possibly contributing to neovascularization during the development of diabetic retinopathy (DR), another common microvascular complication (^18^). Accordingly, various studies have reported the association of polymorphisms in the *ARHGAP22* gene with DR (^14,15,19,20^), probably due to the involvement of this gene in endothelial cell angiogenesis and the augmentation of capillary permeability (^14^). Among these polymorphisms, rs3844492 has been previously associated with a threefold increase in the risk for DR in patients with T2DM (^15^). No study has evaluated the association between the rs3844492 polymorphism and DKD yet.

The *engulfment and cell motility 1 (ELMO1)* gene, located on chromosome 7p14.2-p14.1 (^21^), encodes a member of engulfment and cell motility protein family. This family is involved in various processes, such as apoptotic cell phagocytosis (^22^), fibroblast migration (^23^), cytoskeleton reorganization (^24^), and lymphocyte infiltration (^25^), which are achieved through interaction with the Dock180 protein. Genome-wide association studies have identified polymorphisms in *ELMO1* associated with the development of DKD (^26^). One of the most studied polymorphism in *ELMO1* is rs741301, which seems to be substantial in DKD susceptibility, with the G allele conferring an increased risk for the development of this complication (^27,28^, ^29^).

Thus, given the relevance polymorphisms in the *ARHGAP22* and *ELMO1* genes appear to have in mechanisms related to the development of diabetic chronic complications, and considering no previous study has evaluated the frequency of these polymorphisms in a Brazilian sample, this study aimed to investigate the association between the rs3844492/*ARHGAP22* and rs741301/*ELMO1* polymorphisms and DKD in patients with T2DM.

## METHODS

### Sample description and clinical and laboratory analyses

This case-control study was designed following the STROBE and STREGA guidelines for association studies (^30^, ^31^). The sample consisted of 1,043 patients with T2DM recruited from the outpatient clinic of the Endocrine Division at *the Hospital de Clínicas de Porto Alegre* and the *Grupo Hospitalar Conceição* (Rio Grande do Sul, Brazil) between January 2005 and December 2013 (^32^).

Individuals were classified as having T2DM according to the American Diabetes Association (ADA) guidelines (^1^). The presence of DKD was determined following the Kidney Disease Improving Global Outcomes (KDIGO) recommendations, using urinary albumin excretion (UAE) levels and the estimated glomerular filtration rate (eGFR), which was calculated using the Chronic Kidney Disease Epidemiology Collaboration (CKD-EPI) equation (^33^). Patients were categorized into two groups based on renal function: controls (n = 303), consisting of patients with T2DM for over 10 years and no renal damage (UAE < 30 mg/g and eGFR ≥ 60 mL/min/1.73 m^2^); and two patients with DKD (n = 740, cases), comprising those with moderate DKD (UAE 30 – 300 mg/g and/or eGFR 30 – 59 mL/min/1.73 m^2^, n = 358) or severe DKD (UAE > 300 mg/g and/or eGFR 1 – 29 mL/min/1.73 m^2^, n = 382).

The DR diagnosis was established through direct ophthalmoscopy (fundus eye examination) and classified as absent, non-proliferative DR (NPDR), or proliferative DR (PDR) based on the most severely affected eye, following the scale developed by the Global Diabetic Retinopathy Group (^34^). Ethnicity was self-identified, with 76.9% white patients in the case group and 77.9% in the control group.

A standard questionnaire was employed to gather information on age, age at DM diagnosis, duration of T2DM, and medications used. All patients underwent physical and laboratory examinations, as detailed in a previous study by the group (^35^). Patients were weighed without shoes, in light outdoor clothes, and had their height measured. Body mass index was calculated as weight (kg)/height (meters)^2^. Blood pressure (BP) was measured twice after a 5-minute rest in the sitting position using a digital sphygmomanometer (Omron). The mean value of the two measurements was used to calculate systolic and diastolic BP. Hypertension was defined as BP levels ≥ 140/90 mmHg or if the patient was taking antihypertensive drugs.

Serum and plasma samples were collected after a 12-hour fast for laboratory analysis (^35,36^). Creatinine levels were determined using the Jaffe reaction. Glycated hemoglobin (HbA1c) levels were measured by different methods and the results were traceable to the Diabetes Control and Complications Trial method by off-line calibration or using a conversion formulae (^37^). Triglycerides, total plasma cholesterol, and high-density lipoprotein (HDL) cholesterol were assayed using enzymatic methods, and low-density lipoprotein (LDL) cholesterol was calculated using the Friedewald formula. Albuminuria was determined by immunoturbidimetry (Sera-Pak immune microalbuminuria; Bayer, Tarrytown, NY, USA). All subjects provide assent and written informed consent prior to participation. The study protocol received approval from the Research Ethics Committee of the *Hospital de Clínicas de Porto Alegre* (CAAE No. 63844622.7.0000.5327).

#### Genotyping

The selection of the *rs3844492/ARHGAP22* (A/G) and rs741301/*ELMO1* (C/T) polymorphisms was based on literature studies indicating their association with the development of DM complications (^14^, ^27,29^). Total DNA was extracted from peripheral blood leukocytes using the FlexiGene DNA kit (Qiagen, Hilden, Germany).

Genotyping of the polymorphisms was conducted using the allele discrimination-real-time polymerase chain reaction (PCR) technique. Specific primers and probes for each polymorphism were utilized, contained in the TaqMan SNP Genotyping Assay 20x (Thermo Fisher Scientific, Foster City, CA, USA) (rs3844492/ARHGAP22 – assay ID: C_7547487_20; rs741301/*ELMO1* – assay ID: C__2672066_1_). The real-time PCR reactions were carried out on a 384-well plate, with a total volume of 5 µl per well, using 2 ng of DNA, TaqPath ProAmp Master Mix 1x (Thermo Fisher Scientific) and TaqMan SNP Genotyping Assay 1x. The plates were then placed in a real-time PCR thermocycler (Viia7 Real Time PCR System; Thermo Fisher Scientific) and heated for 10 minutes at 95°C, followed by 40 cycles of 95ºC for 15 seconds and 62°C for 1 minute.

During the experiments, a small number of samples amplified only for one of the polymorphisms analyzed, which is why the number of patients differs between both polymorphisms. For the analyses of rs3844492/*ARHGAP22,* we included 273 controls and 683 cases, while for the analyses of rs741301/*ELMO1*, we included 256 controls and 663 cases.

#### Statistical analyses

Genotype and allele frequencies of the polymorphisms in *ARHGAP22* and *ELMO1* genes were estimated by direct allele counting, and the Hardy-Weinberg equilibrium (HWE) was assessed using the Chi-squared test. Allele and genotype frequencies were compared between groups of subjects using Chi-squared tests. Moreover, genotypes were compared between groups considering additive, recessive, and dominant inheritance models, which were categorized according to a previous study (^38^).

Normal distributions of quantitative clinical and laboratory variables were assessed using the Kolmogorov-Smirnov and Shapiro-Wilk tests. Variables with a normal distribution are shown as mean ± standard deviation (SD). Variables with skewed distribution were log-transformed before analysis and are presented as median (25th – 75th percentile values). Categorical data are expressed as percentages. Clinical and laboratory characteristics were compared between case and control groups, as well as between groups of patients categorized according to different genotypes of the two polymorphisms using appropriate statistical tests, such as Student’s t-test, one-way analysis of variance (Anova) or Chi-squared test.

The magnitude of association between the *ARHGAP22* and *ELMO1* polymorphisms and DKD was estimated using odds ratios (OR) with 95% confidence intervals (CI). Multivariate logistic regression analyses were conducted to evaluate the independent association of each individual polymorphism with DKD, adjusting for possible confounders. Linear regression analyses were conducted to evaluate the independent association of each individual polymorphism with laboratorial markers of DKD (creatinine and albuminuria levels, and eGFR), adjusting for possible confounders. Statistical analyses were performed using the Statistical Package for the Social Sciences (SPSS®), version 18.0 software (Chicago, IL), with p-values < 0.05 considered significant.

## RESULTS

### Sample description

Table 1 details clinical and laboratory characteristics of the control group (patients with T2DM for over 10 years without DKD) and the case group (patients with T2DM and DKD). There were no significant differences between groups regarding ethnicity and mean age (p = 0.790 and p = 0.347, respectively). In the case group, the frequency of men was 52.4% and the duration of T2DM was 18.9 ± 10.4 years, whereas in the control group, the frequency of men was 35.2%, and the duration of DM was 20.5 ± 8.5 (p < 0.001 and p = 0.011, respectively). As expected, there was a higher prevalence of hypertension and DR in patients with DKD compared to the control group (P < 0.0001).

**Table 1:** Clinical and laboratory characteristics of type 2 diabetes mellitus patients without and with diabetic kidney disease

Characteristics	Controls (n = 303)	Cases with DKD (n = 740)	p-value*
Age, years	67.0 ± 10.7	66.3 ± 11.2	0.347
T2DM duration, years	20.5 ± 8.5	18.9 ± 10.4	0.011
Gender, % males	35.2	52.4	< 0.0001
Ethnicity, % non-white	22.1	23.1	0.790
BMI, kg/m^2^	28.8 ± 5.2	28.4 ± 5.2	0.288
HbA1c, %	7.4 ± 1.7	7.6 ± 2.1	0.164
Arterial hypertension, %	79.6	90.6	< 0.0001
Triglycerides, mg/dL	135.0 (95.0-189.0)	157.0 (106.0-231.5)	< 0.0001
Cholesterol total, mg/dL	192.9 ± 46.5	192.1 ± 53.0	0.822
HDL cholesterol, mg/dL	48.2 ± 12.5	43.9 ± 13.6	< 0.0001
LDL cholesterol, mg/dL	110.2 ± 41.8	112.6 ± 45.8	0.486
Diabetic retinopathy, %	42.8	64.3	< 0.0001
eGFR, mL/min per 1.73 m^2^	83.0 (71.0-96.0)	42.0 (13.0-62.0)	-
UAE, mg/g	5.0 (3.0-10.9)	92.5 (28.9-393.6)	-
Creatinine, µg/dL	0.8 (0.7-0.9)	1.4 (1.0-4.3)	-
DM medication, % Met / Ins / Sulf / Met + Ins / Ins + Sulf / Met + Sulf / Met + Sulf + Ins	17.7 / 19.7 / 21.8 / 8.8 / 2.7 / 23.8 / 5.4	9.3 / 47.8 / 16.9 / 12.6 / 1.7 / 10.4 / 1.4	-

Results are shown as mean ± standard deviation, median (25th-75th percentiles) or %.

*p-values were computed using Student’s *t*, or Chi-squared tests, as appropiated.

DKD: diabetic kidney disease; T2DM: type 2 diabetes mellitus; BMI: body mass index; HbA1c: glycated hemoglobin; HDL: high-density lipoprotein; LDL: low-density lipoprotein; eGFR: estimated glomerular filtration rate; UAE: urinary albumin excretion; Met: metformin; Ins: insulin; Sulf: sulfonylurea.

### Genotype and allele frequencies in case and control groups

Table 2 summarizes genotype and allele frequencies of rs3844492/*ARHGAP22* and rs741301/*ELMO1* in cases and controls. Allele frequencies of the rs3844492/*ARHGAP22* polymorphism did not differ between cases and controls (G allele: 37.0% *versus* 38.0%, respectively; p = 0.623). Similarly, no significant difference was observed in the genotype frequencies of this polymorphism between groups (G/G: 15.7% in cases *versus* 16.8% in controls; p = 0.069). This result remained consistent across the recessive, additive, and dominant inheritance models (all p > 0.050). After adjusting for gender, HbA1c, duration of DM, and triglyceride levels, the presence of the G allele was associated with risk for DKD (OR = 1.435, 95% CI 1.023 - 2.011; p = 0.036, for the dominant model).

**Table 2:** Genotype and allele frequencies of rs3844492/*ARHGAP22* and rs741301/*ELMO1* polymorphisms in : type 2 diabetes mellitus patients without and with diabetic kidney disease

	Controls (n =273)	Cases (n = 683)	Unadjusted p-value*	Adjusted OR (95% IC)/p-value†
rs3844492/*ARHGAP22*				
Genotype				
A/A	134 (49.1)	289 (42.3)	0.069	1
A/G	93 (34.1)	287 (42.0)		1.627 (1.120-2.366)/ 0.011
G/G	64 (16.8)	107 (15.7)		1.079 (0.671-1.735)/ 0.754
Allele				
A	0.62	0.63	0.623	
G	0.38	0.37		
Recessive model				
A/A + A/G	227 (83.2)	576 (84.3)	0.724	1
G/G	46 (16.8)	107 (15.7)		0.863 (0.553-1.346)/ 0.515
Additive model				
A/A	134 (74.4)	289 (73.3)	0.789	1
G/G	46 (25.6)	107 (27.7)		1.064 (0.663-1.708)/ 0.797
Dominant model				
A/A	134 (49.1)	289 (42.3)	0.067	1
G/G + A/G	139 (50.9)	394 (57.7)		1.435 (1.023-2.011)/ 0.036
rs741301/*ELMO1*	(n = 256)	(n = 663)		
Genotype				
T/T	87 (34.0)	215 (32.4)	0.800	1
T/C	110 (43.0)	282 (42.5)		1.163 (0.785-1.724/ 0.451
C/C	59 (23.0)	166 (25.1)		1.165 (0.729-1.863)/ 0.523
Allele				
T	0.55	0.54	0.527	
C	0.45	0.46		
Recessive model				
T/T + T/C	197 (77.0)	497 (75.0)	0.587	1
C/C	59 (23.0)	166 (25.0)		1.069 (0.707-1.616)/ 0.752
Additive model				
T/T	87 (59.6)	215 (56.4)	0.577	1
C/C	59 (40.4)	166 (43.6)		1.159 (0.720-1.864)/ 0.544
Dominant model				
T/T	87 (34.0)	215 (32.4)	0.710	1
C/C + T/C	169 (66.0)	448 (67.6)		1.164 (0.809-1.674)/ 0.413

Results are presented as n (%) or proportion.

*p-values were calculated using χ^2^ tests; t p-values and odds ratio (95% confidence interval) obtained using logistic regression analyses adjusting for gender, triglycerides levels, duration of diabetes mellitus and glycated hemoglobin. Genotype distributions of the two polymorphisms were not in Hardy-Weinberg equilibrium.

Regarding rs741301/*ELMO1*, genotype and allele frequencies did not differ significantly between cases and controls (p = 0.800 and p = 0.527; respectively). This polymorphism was also not associated with DKD when analyzing different inheritance models (all p > 0.500). After logistic regression with adjustments for covariates (gender, HbA1c, duration of DM, and triglyceride levels), this polymorphism remained not associated with DKD (all p > 0.400; Table 2).

**Figure 1:**
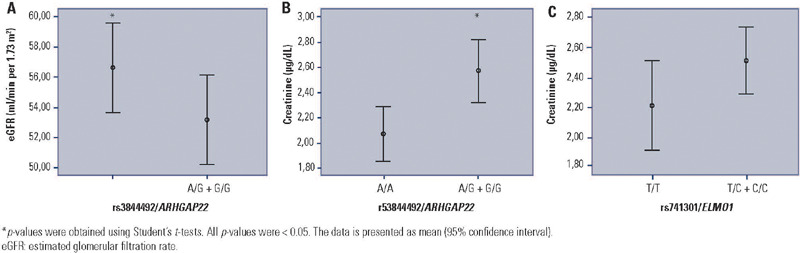
Diabetic kidney disease laboratory markers in patients with type 2 diabetes mellitus according to the rs3844492/ARHGAP22 and rs741301/*ELMO1* genotypes. **(A)** Estimated glomerular filtration rate and **(B)** creatinine levels in patients stratified according to the presence of G allele of the rs3844492/*ARHGAP22* polymorphism (dominant model), and **(C)** creatinine levels in patients according to the presence of the C allele of the rs741301/*ELMO1* polymorphism (dominant model).

In an exploratory analysis, clinical markers associated with DKD were compared after stratifying patients by the presence of the G allele of rs3844492 and C allele of rs741301 under the dominant model. Interestingly, the presence of the G allele of the rs3844492*/ARHGAP22* polymorphism was associated with a decrease in eGFR [A/G+G/G: 56.0 (18.0 – 82.0); A/A: 60.0 (34.0 – 80.0); p = 0.012; Figure 1A] and an increase in creatinine levels [A/G+G/G: 1.20 (0.88 – 2.80); A/A: 1.13 (0.86 – 1.69); p = 0.009; Figure 1B]. These associations were also found after adjustment for gender, duration of DM, and use of ACE inhibitor drugs [decrease in the eGFR (B = −5.87; 95%CI −11.59 – −0,16; p = 0.044); and increase in creatinine levels (B = 0.64; 95%CI 0.15–1.12, p = 0.011)]. Likewise, the presence of the C allele of the rs741301 polymorphism in the *ELMO1* gene presented a tendency to be associated with higher creatinine levels in patients with T2DM, but this did not reach formal statistical significance [T/C+C/C: 1.18 (0.90–2.70); T/T: 1.13 (0.80–2.00); p = 0.064, Figure 1C]. After adjustment for gender, duration of DM, and use of ACE inhibitors drugs, we confirmed the association of the C allele of the rs741301 polymorphism in *ELMO1* with increased creatinine levels (B = 0,85, 95%CI 0.34–1.36, p = 0.001).

*p-values were obtained using Student’s *t*-tests. All p-values were < 0.05. The data is presented as mean (95% confidence interval).

eGFR: estimated glomerular filtration rate.

## DISCUSSION

The *ARHGAP22* e *ELMO1* genes appear to be significant in the pathophysiology of diabetic chronic complications. Studies have reported associations between polymorphisms in the *ELMO* gene and the development of DKD. To date, polymorphisms in the *ARHGAP22* gene have only been analyzed in the context of DR. Therefore, this study investigated the association between the rs3844492 polymorphism in the *ARHGAP22* gene and rs741301 polymorphism in the *ELMO1* gene with DKD in patients with T2DM from a Brazilian population.

Our results, for the first time, revealed that the G allele of rs3844492*/ARHGAP22* polymorphism was associated with risk for DKD. Moreover, the presence of this allele was associated with decreased eGFR values and increased creatinine levels in patients with T2DM. As aforementioned no study has investigated the rs3844492 polymorphism in the *ARHGAP22* gene within a sample of DKD patients to date. Li and cols. (^15^) identified that the rs3844492 and rs10491034 polymorphisms in the *ARHGAP22* gene were associated with DR in patients with T2DM from a Chinese population (rs3844492: OR = 3.52; 95%CI 1.26–9.85; p = 0.011 for the recessive model; rs1049104: OR = 0.47; 95%CI 0.26 0.84; p = 0.009 for the additive model). Further, Han and cols. (20) reported that the rs10491034 G/G genotype was associated with a more advanced stage of DR compared to the G/T genotype, suggesting an additive effect for the number of G alleles on the impact on DR susceptibility. Another study demonstrated that the presence of the C allele of rs4838605/ARHGAP22 was associated with an increased risk of T2DM (OR = 1.57; 95%CI 1.08–2.27; p = 0.018) (^17^). Accordingly, Huang and cols. (^18^) reported that higher *ARHGAP22* expression was positively correlated with the quantity of circulating EPCs in the peripheral blood of patients with T2DM and DR. Therefore, the authors suggested that *ARHGAP22* plays a role in the mobilization and activation of EPCs, which may contribute to neovascularization during the development of DR.

Our findings, which show that the rs3844492/*ARHGAP22* G allele is associated with a worse profile of DKD markers and risk for DKD, align with the aforementioned studies, suggesting that, beyond its involvement in the pathophysiology of DR, the *ARHGAP22* gene may also play a role in the progression of renal damage in patients with T2DM. Considering the shared pathways in the development of DKD and DR, as well as the occurrence of angiogenesis and neovascularization processes in DKD, it is plausible that the *ARHGAP22* gene may influence the renal tissue. This influence could impact the quantity of available EPCs for endothelial repair and the formation of new vessels in the kidney, contributing to a decline in the glomerular filtration process.

We also examined the frequency of rs741301 in the *ELMO1* gene among patients with T2DM, with or without DKD. This polymorphism has been previously linked to DKD in various populations. For instance, Mehrabzadeh and cols. (^28^) reported an association between the rs741301 G allele and risk of DKD (OR = 2.5; 95%CI 1.2 – 5.4; p = 0.010) in Iranian patients with T2DM (^28^). Similarly, the G allele of this polymorphism was found to be associated with risk of DKD in patients with T2DM from a Chinese population (OR = 1.75; 95%CI 1.19–2.28; p < 0.001) (^27^). Moreover, in a case-control study involving patients with T2DM from Egypt, the rs741301 G/G genotype conferred increased risk of DKD (OR = 2.7; 95%CI 1.4–5.3; p = 0.016) (^29^).

In contrast, we did not find any association between the rs741301/*ELMO1* polymorphism and DKD. This finding agrees with two other studies in Egyptian (^39^) and Polish (^40^) populations, which also failed to demonstrate such association. The discrepancies among studies investigating the rs741301*/ELMO1* could be attributed to differences in DKD classification criteria: most studies used the albumin/creatinine ratio (^28,29,39,40^) or focused on the presence of microalbuminuria or serum creatinine levels (^27^). However, our study classified patients according to the KDIGO guidelines (^3^), utilizing both UAE and eGFR values. Although our study did not establish a direct link between the studied polymorphism and

DKD, the presence of the C allele of the rs741301/*ELMO1* polymorphism appeared to be associated with higher creatinine levels; although this association did not reach formal statistical significance. This suggests that this polymorphism might be involved in pathways related to elevated creatinine levels in individuals with DM, rather than directly influencing urine albumin levels. Further research is needed to confirm the impact of this polymorphism on creatinine levels.

In addition to polymorphism analysis, studies have indicated a correlation between the expression of *ELMO1* and renal damage (^41,42^). Hathaway and cols. (^41^) conducted an experimental study involving Akita mice with genetically induced varying levels of *Elmo1* expression, and demonstrated that the severity of renal fibrosis and UAE levels in diabetic mice correlated with *Elmo1* expression levels. Moreover, the levels of reactive oxygen species increased in accordance with the rise in *Elmo1* expression, suggesting the potential involvement of this gene in renal damage, possibly by increased oxidative stress (^41^). Furthermore, an interaction between *ELMO1* and the enzyme cyclooxygenase 2 (COX-2) enhanced the positive regulation of fibronectin, a primary component of the extracellular matrix, leading to COX-2-mediated fibronectin accumulation (^42^). The accumulation of extracellular matrix in the glomerulus is a characteristic feature of many renal diseases, representing a significant lesion in DKD (^42^). Moreover, Sharma and cols. (^43^) identified that *ELMO1* has a protective effect against apoptosis in endothelial cells from the kidneys of both zebrafish and human samples. In contrast, Shimazaki and cols. (^44^) reported increased *Elmo1* expression in the kidneys of diabetic mice compared to control mice, possibly contributing to the development and progression of DKD. This contribution was attributed to the increased expression of TGF-β1, collagen type I, and fibronectin, ultimately leading to the accumulation of proteins in the extracellular matrix.

This study identified associations between the polymorphisms analyzed and changes in laboratory markers of renal damage. However, it is important to carefully interpret the results due to certain limitations. First, the observed genotype frequencies of the rs3844492 and rs741301 polymorphisms deviate from those expected by the HWE in the control group. Despite this, real-time PCR genotyping is a reliable technique, and we rigorously verified all genotype data manually to eliminate possible errors. We also performed duplicate genotyping on 10% of samples to ensure accuracy. Second, the possibility of a type II error cannot be fully excluded in our statistical analyses. Although our study had over 80% power (α = 0.05) to detect an OR ≥ 1.5 for the risk of DKD, and sample sizes were aligned with our calculations, we cannot completely rule out the chance that the rs3844492/*ARHGAP22* and rs741301/*ELMO1* polymorphisms may be associated with DKD at lower ORs.

In conclusion, we demonstrated that the rs3844492 polymorphism in the *ARHGAP22* gene is associated with changes in clinical markers of renal damage (creatinine and eGFR) and with risk for DKD in patients with T2DM from southern Brazil. Moreover, the rs741301 polymorphism in the *ELMO1* gene seems to be associated with creatinine levels in the studied population. It is important to note that no other study has evaluated these polymorphisms in a sample of DKD patients from Brazil.
